# Work attendance anxiety, precarious work schedules, and job satisfaction of essential retail workers during the early COVID-19 pandemic

**DOI:** 10.1371/journal.pone.0318586

**Published:** 2025-03-27

**Authors:** Hyeri Choi, Ioana Marinescu

**Affiliations:** 1 School of Labor and Employment Relations, University of Illinois Urbana-Champaign, Urbana-Champaign, Illinois, United States of America; 2 School of Social Policy and Practice, University of Pennsylvania, Philadelphia, Pennsylvania, United States of America; Institute for Prevention and Occupational Medicine of the German Social Accident Insurance, Institute of the Ruhr University Bochum (IPA), GERMANY

## Abstract

Building upon the Job Demands-Resources model, this study examines the relationships among precarious work schedules, work attendance anxiety, and employer support during the early COVID-19 pandemic. For part-time retail workers, the prevalence of precarious work schedules has been a persistent problem. Additionally, new challenges such as work attendance anxiety and lack of employer support emerged during the COVID-19 pandemic. Data were collected through an online survey on the Amazon Mechanical Turk platform. Between July and August 2020, we secured a sample of U.S. part-time workers in “food and beverage retail stores” (N = 179). Using Ordinary Least Squares regression analyses, we find that work attendance anxiety, lack of control over work hours, and non-standard working hours are negatively associated with job satisfaction. In terms of employer support, personal protective equipment is associated with lower levels of work attendance anxiety and alleviates the impact of work attendance anxiety on job satisfaction. The findings provide valuable insights for employers and managers aiming to enhance job satisfaction and effectively respond to challenges posed by health crises.

## Introduction

COVID-19 exacerbated uncertainties in the workplace, especially for essential workers who were unable to work remotely [1]. Even at the height of the pandemic, the U.S. food retail workers deemed ‘essential,’ such as those in grocery stores, were required to continue working on-site. Part-time retail workers were often given low average hours and pay, while their schedules often fluctuated from week to week without advance notice, making it difficult to predict how much they can earn and work [2–4]. Work hour unpredictability and instability grew as consumer demand surged and subsided, and coworkers fell ill from the virus [5–7]. Although precarious employment is not a new phenomenon, COVID-19 has brought it to the forefront of scholarly and public discourse at a global level [1,8,9].

Perhaps not surprisingly, the COVID-19 pandemic intensified a workplace challenge: anxiety over going to work. Grocery workers had high COVID-19 infection rates, and those who could not practice social distancing at work had significantly higher anxiety or depression [6]. Also, employers varied in their provision of personal protective equipment and paid leave for COVID-related reasons, leaving many retail workers at even greater risk for the coronavirus [10,11]. While work attendance anxiety can occur for various reasons [12,13], the pandemic introduced a new risk of infection, illness or death, heightening perceptions of risk and work-related anxiety. Thus, it is essential to identify employer practices that will improve safety and job satisfaction while reducing workplace anxiety during infectious disease outbreaks.

The Job Demands-Resources (JD-R) model posits that job demands necessitate continuous physical and/or psychological effort, potentially leading to adverse health effects, whereas augmenting job resources can mitigate such demands and prevent unfavorable outcomes. Building upon the JD-R model, the current study examines the relationships among precarious work schedules, work attendance anxiety, employer support, and job satisfaction during the early COVID-19. It is critical for employers to comprehend ways to enhance job satisfaction as job dissatisfaction can result in reduced productivity, increased absenteeism, and higher rates of turnover [14–16]. Also, job satisfaction has profound consequences for workers’ physical, psychological, and family well-being [16–20]. In this study, we draw on original survey data to address the following research questions:

Q1: How are work attendance anxiety and precarious work schedules associated with job satisfaction among essential retail workers who continued working on-site during the COVID-19 pandemic?
Q2: What types of employer support are associated with work attendance anxiety and job satisfaction among essential retail workers who continued working on-site during the COVID-19 pandemic?

Previous studies document the working conditions of essential workers during COVID-19 and their psychological distress such as anxiety, depression, insomnia, and burnout [1,6,21,22]. The current paper extends and updates the literature on job satisfaction among essential workers, drawing on the JD-R model. Moreover, relatively less attention has been paid to work attendance anxiety specifically, as opposed to general mental health or psychological distress, to capture the challenges of workers’ experience [5,23]. In this regard, this study adds to the literature by shedding light on essential retail workers’ job satisfaction by examining both pre-existing problems (precarious work schedules) and new challenges (work attendance anxiety and employer support policies) during the early months of COVID-19 pandemic. The findings will have implications for employers and workers in industries that consistently encounter workplace anxiety and precarious work schedules. They will offer valuable insights into how employers can enhance workplace satisfaction and mitigate workplace anxiety amid external challenges.

This paper proceeds as follows. After a brief discussion of the theoretical framework and relevant literature, we present our empirical approach and results, followed by a discussion and conclusion.

## Theoretical framework

**Fig 1:**
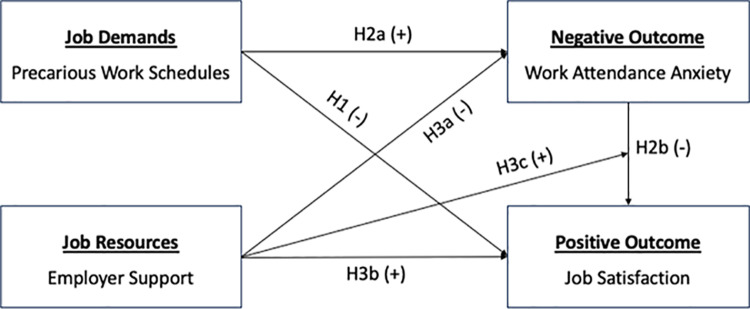
Our conceptual framework. Notes: Constructed by the authors.

The study builds upon the Job Demands-Resources (JD-R) model, which encompasses various factors that influence job-related experiences and outcomes. In this study, we include precarious work schedules, work attendance anxiety, and employer support as key factors within the JD-R framework (Fig 1) [24]. This approach helps us understand how new and existing demands intersect and compound during the COVID-19 pandemic, and to identify strategies employers can implement to provide resources and support.

According to the JD-R model, job demands encompass various aspects of the job that require sustained physical and/or psychological effort, resulting in physiological and/or psychological costs [25]. The study conceptualizes precarious work schedules as job demands, as they make it difficult for workers to predict their earnings and hours, which further leads to work-family conflict and psychological distress [4]. Additionally, the JD-R model emphasizes the importance of job resources as protective factors that can reduce job demands and mitigate negative health consequences [25]. In our study, we explore the role of employer support as job resources, which includes provisions such as personal protective equipment, paid leave, flexible work schedules, and wage increases.

Specifically, we examine the relationship between these job demands and resources, and job satisfaction, i.e., the positive emotional outcome defined as “a pleasurable or positive emotional state resulting from the appraisal of one’s job or job experiences” [26]. Job satisfaction serves as a measurement of employee well-being and is considered a predictor for job performance and engagement [27]. We further explore a negative emotional outcome – work attendance anxiety – which we conceptualize as a psychological cost resulting from job demands.

Since this was measured during the COVID-19 pandemic, it is challenging to distinguish between the demands posed by the pandemic situation and the general workplace anxiety experienced by workers, as they are closely associated. However, the influence of COVID-19 may have heightened work attendance anxiety due to the increased health risks and uncertainties associated with the pandemic. Specifically, the health risks workers are exposed to on the job may be conceptualized as a job demand that is unique to the pandemic period. This context justifies considering work attendance anxiety within our analysis, as it reflects the unique psychological costs imposed by the pandemic on essential workers. Empirical research on JD-R model suggests a negative association between job demands and job satisfaction, and a positive association between job demands and work attendance anxiety [28,29]. Conversely, there is a positive association between job resources and job satisfaction, and a negative association between job resources and anxiety.

We conceptualize job satisfaction as a result of all job aspects, including work attendance anxiety. The availability of job resources has the potential to buffer the negative impact of work attendance anxiety on workers’ well-being. Consequently, we also examine whether employer support during COVID-19 mitigates the negative impact of work attendance anxiety on job satisfaction. Our framework aims to provide a comprehensive understanding of how various aspects of jobs interact to influence employee well-being, particularly in the context of a global health crisis.

## Literature review and hypothesis development

### U.S. food retail workforce and job satisfaction

U.S. food retail workers are among the most visible essential workers in “food and beverage retail stores”, including workplaces such as grocery stores, convenience stores, and seafood markets. This workforce does not include restaurant workers. The food retail industry in the U.S. employs approximately 3.3 million people as of May 2024, with key occupations including cashiers, stock clerks and order fillers, and first-line supervisors/managers of retail sales workers [30,31]. The average hourly earnings for all employees in the food retail sector were $21.47, with average weekly hours at 28.7 [30]. During the COVID-19 pandemic, this industry faced significant challenges, including fluctuating demand and the necessity for enhanced safety measures, which impacted job conditions and worker safety [30–32]. The industry’s critical role as an essential service was highlighted as workers continued to provide essential goods under challenging circumstances. The purpose of this study is to understand the job satisfaction of U.S. food retail workers who faced two layers of risk at the same time: precarious employment and the COVID-19 pandemic [9–11]. Job satisfaction is a critical component of workers’ well-being and engagement. Pre-COVID-19 studies demonstrated that working conditions, earnings, and organizational support are important elements of U.S. retail workers’ job satisfaction [33–35]. Following COVID-19, a few studies documented a decline in job satisfaction across different workforces [14,22,36].

### Existing problem: Precarious work schedules

One problem that has persisted during the pandemic is the prevalence of precarious work schedules, which is associated with lower job satisfaction. Unstable and unpredictable work schedules are common in the retail sector. The majority of part-time retail workers experience work-hour fluctuations and know their work schedule one week or less in advance [3]. Studies show that these workers continue to be subject to precarious work schedules during COVID-19 [1,7,9]. Workers who remained employed during this time experienced more precarious work, predicting lower fulfillment of survival needs over time [1].

Prior literature suggests that precarious work schedules have four dimensions: instability of hours (work-hour fluctuations), the timing of hours (starting and finishing times – standard vs. non-standard hours), predictability of hours (advance notice, last-minute schedule changes), and schedule control (worker input into the number and timing of hours) [3,37]. These dimensions can be understood in two ways: procedural fairness and outcome favorability. There is a growing understanding among organizational practitioners that workers’ job attitudes and behaviors are both influenced by the fair processes and favorable outcomes of work-related decisions [38]. When workers are given little control over their schedule (process) and do not have their preferred shifts (outcome), they are less satisfied with their jobs than those with preferred and flexible schedules [37,39,40]. The unpredictable nature of these types of schedules can be very stressful and require workers to constantly adjust their lives to accommodate the changing hours. This constant adaptation can lead to feelings of powerlessness and frustration, which can lead to psychological costs like low job satisfaction. Thus, in line with the JD-R model and prior literature, we consider precarious work schedules as job demand and hypothesize as the following:

H1. Precarious work schedules – lack of control over work hours, unpredictability in work schedules, work hours instability, and non-standard work hours– will be associated with lower levels of job satisfaction.

### Emerging problems: Work attendance anxiety and employer support

#### Work attendance anxiety.

An emerging problem during the early COVID-19 pandemic was work attendance anxiety. Workers in the food retail, food service, and hospitality industries experienced higher levels of anxiety because of the many uncertainties and personal safety issues during COVID-19 [23]. Available studies document the prevalence of general anxiety during COVID-19, but workplace anxiety specifically was not sufficiently addressed [22,41].

Investigating workplace anxiety is important as unmanaged anxiety can lead to long-term negative effects on work performance, motivation, health, and job satisfaction [12,42–45]. Despite its significant consequences, to date, workplace anxiety has not been adequately addressed as a health aspect in organizational psychology models. There have been few studies investigating workplace anxiety in organizational settings, and there are no unified terms or definitions. Muschalla and Linden (2009) [13] uses the term ‘workplace phobia’ and defines it as a “phobic anxiety reaction with symptoms of panic occurring when thinking of or approaching the workplace.” Jones et al. (2016) [12] measures job anxiety using three anxiety states by asking how much of the time the job made them feel tense, worried, or uneasy. Different types of stressors can lead to various dimensions of job anxiety.

Studies have shown that workplace phobia can be empirically distinguished from conventional anxiety disorders through psychological and physiological responses to work-related stress. One experimental study identified individuals with workplace phobia by their intense fear and severe anxiety symptoms when approaching the workplace, along with higher heart rate and subjective reports of fear [46]. Further research indicated that while some individuals experience both non-work-related anxiety disorders and workplace phobia, workplace phobia is distinct among anxiety sufferers, with some individuals experiencing only workplace phobia without other non-work-related anxiety disorders [13].

In this study, we focus on anxiety over going to work and coin this as “work attendance anxiety.” We define it as “a state of feeling nervous, anxious, or on edge to come into work.” We consider work attendance anxiety as a potential indicator of threats and problematic situations at work, which adversely affects workers’ judgments and their level of job satisfaction [42–44,47]. Also, the unpredictable and uncontrollable nature of precarious work schedules can lead to psychological impacts such as increased work attendance anxiety. Thus, given the limited elaboration on workplace anxiety in existing literature, we study the relationship between precarious work schedule and work attendance anxiety as well as the association between work attendance anxiety and job satisfaction. We hypothesize as the following:

H2a. Precarious work schedules – lack of control over work hours, unpredictability in work schedules, work hours instability, and non-standard work hours– will be associated with higher levels of attendance anxiety.
H2b: Work attendance anxiety will have a negative relationship with job satisfaction.

#### Employer support.

Many essential workers in frontline jobs were not able to access benefits such as personal protective equipment (PPE), paid sick leave, wage increases or flexible work schedules that can help them manage job stress and protect their health [10,11,23,48,49]. The Centers for Disease Control and Prevention guidelines for workplaces on preventing infections carried neither statutory nor regulatory power, leading to substantial variation in safety practices across states, sectors, and firms [5,50]. In such conditions, workers were even less likely to be able to satisfy their psychological and basic survival needs.

Studies demonstrate that employer support and policies can play a significant role in the psychological well-being of their workers. Essential workers who felt protected by employers or governments during COVID-19 experienced a decrease in general anxiety symptoms [5,22,29]. For instance, workers whose employers offered paid leave were less likely to work when sick and more satisfied with their jobs [51]. Under the JD-R framework, we expect that employer support as job resources will help reduce attendance anxiety and buffer the negative impact of attendance anxiety on job satisfaction. Thus, we hypothesize as the following:

H3a: Employer support – PPE, flexible work schedule, wage increase, paid leave – will be associated with lower levels of attendance anxiety.
H3b: Employer support – PPE, flexible work schedule, wage increase, paid leave – will be associated with higher levels of job satisfaction.
H3c: Employer support – PPE, flexible work schedule, wage increase, paid leave – will moderate the relationship between attendance anxiety and job satisfaction.

## Materials and methods

This study employed a correlational cross-sectional study design. We collected data through an online survey on the Amazon Mechanical Turk platform between July and August 2020. We limit the study period between July and August 2020 because the purpose of the current study is to observe the immediate effects of the COVID-19 pandemic. Drawing on original survey data, we conducted Ordinary Least Square (OLS) regression analyses with a set of covariates using survey weights derived from the Current Population Survey (CPS).

### Sample and data

#### Sample inclusion criteria.

First, individuals were required to be part-time hourly workers who were currently employed, had been working since June 2020 or before, worked less than 35 hours a week, and were paid hourly wages. Workers needed to have worked for at least a month to answer the survey questions. Furthermore, workers had to be in non-managerial positions. This excluded self-employed workers and salaried workers (non-hourly workers). We chose to focus on part-time hourly workers because they often experienced more vulnerability related to precarious work schedules and may have received less employer support compared to full-time or salaried employees. This group was particularly relevant to our research objectives. Second, individuals were required to work in “food and beverage stores” within the retail sector. Food and Beverage Stores were classified as NAICS 445: 4971 Supermarkets and other grocery (except convenience) stores, 4972 Convenience Stores, 4980 Specialty food stores, and 4990 Beer, wine, and liquor stores. We focused on food and beverage stores since they had been less impacted by the immediate closures of non-essential businesses in many states. The U.S. government categorized these businesses as essential, so many food and beverage stores remained open. Lastly, we targeted the U.S. working-age population, but the minimum required age for MTurk workers was 18. So, participants were required to be between the ages of 18 and 64.

#### Sample recruitment.

We used Amazon Mechanical Turk (MTurk) platform linked with Qualtrics to recruit respondents in a timely manner. Despite MTurk not always being the optimal method for data collection, its use was particularly valuable given the constraints during the pandemic. It allowed us to reach specific workers effectively, considering their unique schedules, risks, and demands at that time. We conducted qualification tests twice to ensure that respondents met all the sample inclusion criteria. The main survey was conducted between August 6 and August 19, 2020, and 230 workers took the survey among 316 qualified workers (response rate: 72.7%). A small sample size can reduce statistical power and may underestimate the impact of the variables under investigation. However, it is worth noting that the part-time retail workers in food and beverage stores, meeting our inclusion criteria, constituted only a minuscule fraction of the population, representing only 0.04% of the July-August 2020 Current Population Survey (CPS) basic monthly sample. This limited representation underscores the challenge of obtaining a sizable sample from this specific target population.

#### Protection of human subjects.

The researcher protected the participants in the study by adhering to ethical and legal guidelines from the university institutional review board. Before the participants proceeded with the survey, written informed consent was presented through MTurk and obtained by checking the “I agree” box online. The informed consent indicated that participation in the study was voluntary and that participants had the right to withdraw from the study without incurring adverse consequences. The researcher did not collect any personally identifiable information from the participants. Their Worker IDs were only used to pay them correctly and were permanently deleted from the experimenter’s data after payment was complete. Upon completion of the survey, all participants were assigned unique identifying numbers for the purpose of analysis. Access to survey data during collections was restricted via a university-owned and password-protected Qualtrics account, accessible only to co-investigators of the study. Once collection rounds closed, exported data were stored in an encrypted folder on the researcher’s computer.

#### Sampling bias and re-weighting.

MTurk workers tend to be younger, make lower than average income, and be Caucasian [52]. To better approximate a nationally representative sample of individuals, we restricted the CPS Basic Monthly data from July to August based on our sample criteria and use weights based on gender, education, race, and age. All regression models were estimated with these survey weights.

#### Data.

The total sample in the original dataset consisted of 230 participants. Based on the eligibility criteria, the study excluded participants who were not retail workers, such as Instacart personal shoppers, provided inconsistent data (i.e., the greatest number of work hours was smaller than the smallest number of work hours), were below 18 or above 64 years of age, and worked 35 hours or more. The final analytic sample included 179 participants who provided information on all variables used in the analysis. Table 1 provides the individual demographic and socioeconomic characteristics of the sample with comparisons to the CPS in July-August 2020, where the CPS sample was restricted to those workers who met the same eligibility criteria as our study sample. Most participants in our sample were White (74.9%), not married or not partnered (62.6%), and non-immigrants (96.6%). Their mean age was 31.6, 50.8% had not completed college, and 80.4% had an annual household income below $75,000 (Table 1). Our sample was younger, more educated, and had lower household income than the CPS sample (In the CPS sample, college educated workers account for only 9.7%, which is much lower than in our study sample. We have verified that these CPS numbers are reliable by comparing our results with a study that closely aligns with our target industry and study period, utilizing an April 2020 CPS sample. Cho and Lee (2020) [53] reports that 14% of grocery store workers held bachelor’s degrees or higher. It is important to note that this percentage encompasses both part-time and full-time employees. In our CPS sample, we narrow the focus to part-time workers, which may explain why the educational attainment level is lower than in Cho and Lee (2020) [53]).

**Table 1:** Demographic characteristics of workers in MTurk versus Current Population Survey (CPS) sample.

Measurements	MTurk	CPS
**Gender**		
Female	50.8%	56%
Male	49.2%	43%
**Race**		
White	74.9%	83.3%
Black or African American	8.9%	11.8%
Asian	11.1%	4.5%
American Indian or Alaska Native	1.1%	0.5%
Others	3.9%	0.0001%
**Education**		
College and above	49.2%	9.7%
Less than college	50.8%	90.3%
**Age**		
Mean (SD)	31.6 (9.5)	31.5 (14.4)
**Age (by categories)**		
18-27	39.7%	63.9%
28-37	38.5%	12.1%
38-47	15.6%	6.4%
48-57	2.8%	13.8%
58-64	3.4%	3.8%
**Household Income**		
75,000 and above	19.6%	52.3%
Below 75,000	80.4%	47.7%
**Marital Status**		
Married or partnered	37.4%	18.5%
Not Married or not partnered	62.6%	81.5%
**Number of Children**		
0	60.3%	85%
1 and more	39.7%	15%
**Immigrant**		
Immigrant	3.4%	9.1%
Non-immigrant	96.6%	90.9%
Observations	179	297,167

*Source:* Survey data collected by the authors on Amazon Mechanical Turk in July-August 2020 and CPS basic monthly data (July and August 2020).

*Notes:* CPS sample was weighted by the final person-level weight that should be used in analyses of basic monthly data. Original sample size before weighting is 406. CPS sample is restricted to those workers who meet the same eligible criteria as our study sample.

### Measures

#### Precarious work schedules.

Following the measurements of National Survey on Work Schedules, work-hour instability was calculated by dividing the hour range by the reported usual hours [(greatest number of hours worked in a week in the last month – smallest number of hours worked in a week in the last month) ÷ usual work hours in the last month].

For timing of hours, standard hours were a regular day shift and non-standard hours include an evening shift, a night shift, a rotating shift, a split shift, or irregular schedule. We created a dichotomous variable indicating workers who had (1) and had not (0) worked in nonstandard timing.

Predictability of hours was measured by the length of advance notice given to workers regarding their schedules and was categorized as the following: (1) A day or less in advance, (2) 2-6 days in advance, (3) 1-2 weeks in advance, (4) 3 or more weeks in advance, and (5) Schedule never changes. We created a dichotomous variable indicating workers who received their schedule notice in less than a week (1) and a week or more in advance (0).

For schedule control of hours, we asked workers if their total number of work hours each week and starting and finishing time were decided by (1) their employers, (2) their employer, but with employee’s input, (3) employees with certain limits, or (4) exogenous factors that are outside of employees’ and employers’ control. We created a dichotomous variable indicating workers who had no input (1) and some input (0) in their schedule decision.

#### Work attendance anxiety.

Attendance anxiety was measured by asking respondents if they feel nervous, anxious, or on edge to come into work on a 5-point scale. (1 = very slightly or not at all; 2 = a little; 3 = moderately; 4 = quite a bit, 5 = extremely).

#### Job satisfaction.

Job Satisfaction was measured by the Job Satisfaction Index (JSI) scale, which was developed by Brayfield and Rothe (1951) [54]. The JSI comprises 18 items to measure overall job satisfaction (e.g., ‘My job is usually interesting enough to keep me from getting bored’). Job satisfaction can be considered as a “global construct” by combining all feelings and cognitions toward the job. Job satisfaction can also be measured by various “facet items/scales” such as job task, payment, promotion, supervision, or coworkers [27]. The global construct is more appropriate when job satisfaction is considered for the well-being of employees. Facet scales may omit some areas that may be important to the individual. Moreover, simply adding facets for all people may not capture the unique individual methods of combining components to arrive at a summary feeling [55]. Therefore, the study employed a global construct approach to reflect the general job satisfaction of the individuals rather than adding various facets of jobs. Responses followed a five-point Likert scale for each statement ranging from a value of 1 for ‘strongly disagree’ to 5 for ‘strongly agree’. The Cronbach’s alpha equaled.94 in our study.

#### Control variables.

We controlled for gender, age, marital status, household income, and number of children which have been shown to influence job satisfaction [56–59]. Age was calculated by subtracting the birth year and month from the year and month of the survey. Gender was coded as male (0) and female (1). Marital status was categorized as workers who are (1) and are not (0) married or partnered. Number of children was a dichotomous variable indicating workers who have 1 and more children (1) and no children (0). Total household income was coded as below (1) and above (0) $75,000 which was the U.S. median household income.

### Empirical strategy

For the analysis, Ordinary Least Square (OLS) regression analyses were conducted with a set of covariates. We estimated regression models by the following equation:


$$Y_{i}=\beta P_{i}+\gamma X_{i}+\varepsilon_{i}$$

Where *Y*_*i*_ is the job satisfaction or work attendance anxiety for an individual i, *P*_*i*_ are the predictor variables which include four dimensions of precarious work schedules (lack of control over work hours, unpredictability in work schedules, work hours instability, and non-standard work hours) and four types of employer support (PPE, flexible work schedule, wage increase, paid leave). The set of controls, *X*_*i*_ are total household income, marital status, number of children, gender, race, and age. To estimate the relative contribution of each predictor and secure enough observations for each variable, dichotomous variables were used for the analytic purpose. *ε_i_* is the error term. We began by regressing precarious work schedules on work attendance anxiety. Next, we regressed both precarious work schedules and attendance anxiety on job satisfaction, conceptualizing job satisfaction as a result of all job aspects, including work attendance anxiety. Subsequently, we regressed employer support on work attendance anxiety and job satisfaction separately. Finally, we examined the interaction between work attendance anxiety and employer support on job satisfaction to determine if employer support mitigated the negative impact of attendance anxiety on job satisfaction during COVID-19.

## Results

### Descriptive results

Table 2 presents the descriptive statistics for the main variables in our study. Food retail workers reported moderate levels of job satisfaction and attendance anxiety. The mean job satisfaction score was 51.1, ranging from 22 to 81 points, with workers able to report a minimum of 18 and a maximum of 90. The mean attendance anxiety score was 2.7, with a standard deviation of 1.3, indicating moderate anxiety levels among workers and considerable variability in individual experiences. The precarity of work schedules was prevalent. More than half (51.4%) of the workers were working non-standard hours, with the rest working regular day shifts. The average work hour instability ratio was 0.4, meaning that workers’ hours varied by 40% of their usual weekly hours on average. Procedural fairness for work schedule decisions was uncommon. More than half (50.8%) of the workers were notified of their work schedules less than a week in advance. Workers had very little control over their work schedules, with more than half reporting no input at all (61.5%).

**Table 2:** Descriptive statistics: Job satisfaction, work attendance anxiety, and precarious work schedules.

Measures	Percentage or Mean
**Job Satisfaction**	
Mean (SD)	51.1 (13.5)
**Work Attendance Anxiety**	
Mean (SD)	2.7 (1.3)
**Timing of hours**	
Standard hours	48.6%
Nonstandard hours	51.4%
**Predictability of hours**	
1 or more weeks in advance notice	49.2%
Less than a week notice	50.8%
**Schedule Control over Work Hours**	
Some employee input	38.5%
No employee input	61.5%
**Work Hour Instability**	
Mean (SD)	0.4 (0.4)
Observations	179

*Source:* Survey data collected by the authors.

Table 3 describes employer support for food retail workers based on work attendance anxiety level since our study aims to explore the impact of employer support on this anxiety. Overall, 40.2% of workers received PPE, 32.4% received flexible work schedules, and one-fifth had paid leaves and increased hourly wages. Workers with higher anxiety were less likely to be provided with paid leaves (14%) and PPE (76.3%) than workers with lower anxiety (33.7%, 93%, respectively) (p < .05). Notably, our findings indicate that bonus pay and schedule flexibility did not significantly reduce anxiety, despite the common belief that financial incentives would ease workers’ anxiety, especially during the financially strained summer months of the pandemic. This contrast underscores the importance of tangible health and safety measures like PPE over monetary incentives in mitigating anxiety for essential workers who continued to work on-site during the early stages of the COVID-19 pandemic.

**Table 3:** Descriptive statistics: Employer support by work attendance anxiety.

Measures	Lower Anxiety	Higher Anxiety	Overall	Significance
**Employer Support [Multi-select questions]**				
Provided flexible work schedules per worker’s request	32.6%	32.3%	32.4%	
Provided paid leaves if a worker was tested positive or have been directed	33.7%	14.0%	23.5%	***
Provided personal protective equipment	93.0%	76.3%	40.2%	***
Provided special bonus or increased hourly wages	20.9%	20.4%	20.7%	
Others	1.2%	2.2%	1.7%	
Observations	86	93	179	

*Source:* Survey data collected by the authors.

*Notes:* Lower anxiety levels are defined as anxiety scores below 2.66 (mean) whereas higher anxiety levels are defined as scores of 2.66 and above. Two-tailed t-tests were conducted to compare means or percentages between workers with higher and lower anxiety. * p < .05; **p < .01; ***p < .001

### Regression results: The impact of attendance anxiety and precarious work schedules on job satisfaction

Table 4 presents the regression results examining the associations between job satisfaction, attendance anxiety, and precarious work schedules. First, we hypothesized that precarious work schedules will be negatively associated with job satisfaction (H1). Among the four dimensions of precarious work schedules, working non-standard hours and having no control over hours showed a significant negative relationship with job satisfaction (p < .05), partially supporting our hypothesis 1. Workers who had non-standard work shifts reported 3.8 points lower job satisfaction than those with standard hours. Workers who had no input in their work schedule reported 7.4 points lower job satisfaction than those who had some input. This is quite substantial when considering our sample range of job satisfaction was 22 to 81 points.

**Table 4:** Regression results: Association between job satisfaction, and work attendance anxiety, and precarious work schedules.

Measures	Work Attendance Anxiety	Job Satisfaction
**Work Attendance Anxiety**		-3.683***
		(0.630)
**Timing of Hours**		
**Non-standard hours**	0.196	-3.843**
	(0.188)	(1.522)
**Predictability of Hours**		
**Less than a week notice**	0.267	1.339
	(0.207)	(1.687)
**Schedule Control of Hours**		
**No employee input**	-0.052	-7.449***
	(0.194)	(1.578)
**Work Hour Instability Ratio**	0.149	2.267
	(0.169)	(1.430)
Observations	179	179
Adjusted R^2^	0.035	0.354
Demographic controls	Yes	Yes

*Source:* Survey data collected by the authors.

*Notes:* Standard errors are presented in parentheses. Demographic control variables include household income, marital status, number of children, sex, race, and age. * p < .05; **p < .01; ***p < .001

We hypothesized that precarious work schedules will be positively associated with attendance anxiety (H2a). Hypothesis 2a was not supported, as none of the four dimensions demonstrated a statistically significant association with work attendance anxiety. Furthermore, we hypothesized that anxiety about coming to work during the COVID-19 pandemic will have a negative relationship with job satisfaction (H2b). Increased levels of work attendance anxiety were associated with decreased levels of job satisfaction (p < .05). A one-unit increase in attendance anxiety was associated with 3.6 points decrease in job satisfaction. Our result supports the hypothesis. Please see S1 Appendix in S1 File A for detailed regression results that present the relationships between demographic variables and job satisfaction.

### Regression results: The impact of employer support on work attendance anxiety and their interaction effects on job satisfaction

Table 5 documents the results examining the associations between employer support, work attendance anxiety, and job satisfaction. We hypothesized that employer support would alleviate work attendance anxiety (H3a). Our results partially support this hypothesis. Among employer support variables, only personal protective equipment (PPE) had a significant association with work attendance anxiety. This relationship was significant even after controlling for demographics (Model 1). However, hypothesis 3b was not supported since none of the employer supports showed statistically significant impact on job satisfaction (Model 2). These results suggest that attendance anxiety may be specifically linked with the health risks of contracting COVID-19, and that these risks could not be well compensated by supports that do not directly address the risk of infection.

**Table 5:** Regression results: Association between work attendance anxiety and employer support.

Outcome Variable			
Measures	Model 1	Model 2	Model 3
	Work Attendance Anxiety	Job satisfaction	Job Satisfaction
**Employer Support**			
PPE	-0.883***	-1.271	-23.715***
	(0.277)	(2.691)	(7.255)
Paid Leave	0.268	-3.791	–
	(0.268)	(2.607)	
Wage Increase	-0.295	2.904	–
	(0.262)	(2.552)	
Flexible Work Schedule	-0.041	3.593	–
	(0.225)	(2.193)	
**Interaction Effect**			
PPE X Work Attendance Anxiety			5.684***
			(2.108)
Work Attendance Anxiety			-8.921***
			(1.973)
Observations	179	179	179
Adjusted R^2^	0.073	0.124	0.291
Demographic Controls	Yes	Yes	Yes

*Source:* Survey data collected by the authors.

*Notes:* Standard errors are presented in parentheses. Control variables include the demographic characteristics and precarious work schedules. * p < .05; **p < .01; ***p < .001

Further, we hypothesized that employer support would moderate the relationship between attendance anxiety and job satisfaction (H3c). The interaction effect between PPE and anxiety was significant, suggesting that the impact of attendance anxiety on job satisfaction varied by PPE support (Model 3). For every 1-unit increase in the anxiety level, workers who did not receive PPE should expect to have 8.9 points lower job satisfaction, while workers who received PPE should only expect to have 3.3 points lower job satisfaction. Our results support the hypothesis 3c.

One might suggest that the positive effect of employer support on job satisfaction is mediated by a reduction in work attendance anxiety. Our mediation analysis indicates that work attendance anxiety partially mediates the relationship between two types of employer support—PPE (*β* = 2.16, p < 0.05) and paid leave (*β* = 1.90, p < 0.05)—and job satisfaction. Sobel’s test confirms the significance of this mediation (see Table C1 in S1 Appendix C in S1 File). The findings highlight the importance of employer support in mitigating work attendance anxiety, which positively influences job satisfaction.

## Discussion

The present study examined the impact of precarious work schedules, work attendance anxiety, and employer support on job satisfaction among essential retail workers who continued working on-site during the early months of the COVID-19 pandemic. The study drew on the Job Demands-Resources (JD-R) model and original survey data to answer these research questions, with the ultimate goal of identifying employer practices that can improve worker well-being and health. By assessing work attendance anxiety, we acknowledged the unique challenges faced by workers during the COVID-19 pandemic. This approach highlights the additional psychological burden and effort required to fulfill job obligations in the face of high-risk situations. The findings of the study suggest that work attendance anxiety is negatively associated with job satisfaction. This result is consistent with prior research that has linked anxiety and other forms of psychological distress to lower job satisfaction and well-being among workers [22,43,45]. Our results demonstrate that work attendance anxiety explained additional variance in job satisfaction above and beyond precarious work schedules.

The study also found that precarious work schedules, such as employer-driven control over work hours and non-standard hours, were negatively associated with job satisfaction. This result aligns with prior research that has related precarious work schedules to lower job satisfaction and well-being among workers [2–4,40]. While predictability and instability were not significant predictors of job satisfaction in this study, these factors may have been typical for these types of jobs even before the pandemic [3,40]. These results highlight the importance of providing retail workers with preferred work schedules that can help improve overall job satisfaction. Employers should consider providing more stable work schedules that are predictable and reliable for their workers, particularly when unexpected opportunities and threats arise in the workplace. Some might suggest a path analysis that precarious work schedules can lead to higher anxiety and thereby leading to lower job satisfaction. However, this was not supported in our analyses (see Table C2 in S1 Appendix C in S1 File).

In terms of employer support, the study documented that workers with higher work attendance anxiety were less likely to be provided with paid leave and PPE than workers with lower anxiety. This result was particularly concerning, as essential retail workers who were unable to practice social distancing at work were at a higher risk of contracting the virus and experiencing negative health outcomes. These findings suggest that employers should have considered prioritizing the provision of paid leave and PPE for essential retail workers, particularly those who were experiencing high levels of work attendance anxiety.

Finally, the study demonstrated that employer support through PPE moderated the relationship between work attendance anxiety and job satisfaction. The negative impact of attendance anxiety on job satisfaction was smaller among food part-time retail workers who received PPE. These findings highlight the importance of providing essential retail workers with adequate protective equipment to help reduce their risk of exposure to the virus and alleviate their anxiety about going to work. This result is consistent with prior research that has identified employer or organizational support as a critical support system in helping workers achieve positive emotions when facing anxiety-provoking situations [5,12].

It is worth noting that in our study, only PPE (not paid leave, wage increase, and flexible work schedule) was found to be associated with work attendance anxiety. This finding raises some interesting questions that warrant further investigation. For instance, one possible explanation could be related to the timing of data collection, which occurred in August 2020, when the pandemic was still raging in many parts of the world. It is possible that PPE was viewed as one of the most critical needs for these essential workers at that time, and the lack of PPE for some workers may have contributed to their anxiety. However, there is a possibility that these patterns may have changed as the pandemic waned. Thus, future studies should revisit this question and see what type of employer support enhances job satisfaction.

Unique situational factors may have rendered other types of precarious work schedules and employer support statistically insignificant in this study. For instance, the pandemic and summer timing meant younger workers had fewer additional demands (academic, social, other work commitments), which could have made the effects of predictability of hours and work hour instability less significant [60] Also, job satisfaction may have been elevated due to the respect and solidarity workers experienced from being deemed essential during the COVID-19 pandemic. Increased social capital can enhance satisfaction and quality of life, potentially mitigating the adverse effects of precarious work schedules during the pandemic [61]. These factors underscore the importance of considering context-specific influences when evaluating job satisfaction.

### Limitations and future research

Our study is not without limitations. The findings were based on descriptive correlational evidence and did not establish a causal relationship between job satisfaction, attendance anxiety, and employer support. Furthermore, the results of the study were specific to scheduling and employer support during the early COVID-19 pandemic. The unique conditions of the pandemic likely amplified the perceived importance of PPE due to the heightened health risks. This focus on PPE may have overestimated its impact on reducing attendance anxiety, while underestimating the effectiveness of other forms of employer support that did not directly mitigate infection risk. Thus, future studies should examine whether these patterns have persisted or changed as the pandemic has progressed and whether similar patterns exist in other industries or job types. Furthermore, future research should investigate the effectiveness of various types of employer support in promoting worker well-being and productivity in the context of both crisis and non-crisis situations.

Additionally, due to the cross-sectional study design, it was challenging to determine whether job satisfaction during the pandemic was higher or lower than pre-pandemic levels. It is possible that job satisfaction has declined during the pandemic. To address these limitations, future studies should conduct follow-up surveys after the pandemic to compare results and provide a more complete understanding of the impact of pandemics on job satisfaction and attendance anxiety.

As previously mentioned, food retail workers experienced fear of COVID-19 transmission. Our study did not directly measure ‘exposure to COVID-19 infection’ as a job demand. This exposure can be a factor leading to workplace anxiety and ultimately to lower job satisfaction. However, we did measure the impact of supports that specifically address this job demand, such as PPE, and demonstrated their importance for the outcomes. This indicates that attendance anxiety is closely linked with the health risks associated with COVID-19, which could not be fully mitigated by supports unrelated to infection prevention. Furthermore, although we did not directly measure the fear of COVID-19 infection, we inquired whether employees were calling out sick or afraid to come into work, which can imply a fear of transmission. Using this proxy, our regression results showed that fear of transmission was negatively associated with job satisfaction, similar to work attendance anxiety (see S1 Appendix D in S1 File).

Two major concerns for MTurk are representativity and measurement error. Respondents recruited online through MTurk are not representative of the general population. Compared to CPS data, our sample was younger and more educated. To address this limitation, the study used CPS weights. Regarding measurement error, we are generally concerned about MTurk workers’ inattention. One of the strategies is to have attention check questions or detailed short answers. In our study, we used an attention check question, and all participants passed the question.

## Conclusion

Our study highlights that anxiety over going to work was an important driver of job satisfaction in addition to the absence of schedule control. Our findings suggest that, under some circumstances, employers can better support workplace satisfaction and reduce anxiety through health and safety related supports rather than financial incentives. During the early stages of the COVID-19 pandemic, providing personal protective equipment was particularly effective in reducing work attendance anxiety and enhancing job satisfaction. These insights are valuable for various workplace contexts where safety, predictability, and satisfaction are concerns. Identifying strategies that can alleviate attendance anxiety and enhance job satisfaction will enable employers to effectively support essential workers’ psychological and physical well-being in times of crisis and beyond.

## Supporting information

S1 File Additional results. (DOCX)
