# Supplementary material for: Work attendance anxiety, precarious work schedules, and job satisfaction of essential retail workers during the early COVID-19 pandemic
Source: PLoS One. 2025 Mar 27;20(3):e0318586. doi: 10.1371/journal.pone.0318586 (PMC11949338; doi:10.1371/journal.pone.0318586)
Supplement: S1 File — (DOCX) [file pone.0318586.s001.docx]

**Work attendance anxiety, precarious work schedules, and job satisfaction of essential retail workers during the early COVID-19 pandemic**

**Supplementary File**

Hyeri Choi and Ioana Marinescu

**S1 Appendix A. Full regression results with demographic characteristics**

Gender, age, education, marital status, and number of children have been shown to influence job satisfaction. Previous studies of job satisfaction and gender showed mixed results, with a substantial amount of research finding that women have higher job satisfaction than men, and some other studies finding no difference between men and women in job satisfaction, particularly when controlling for specific job variables (1,2). Women showed higher job satisfaction than men when viewed collectively but showed different levels of job satisfaction by marital status and number of children. When marital status was considered, one large-scale study found that married women had higher levels of job satisfaction than both married men and unmarried women while the latter two groups had similar levels of job satisfaction (3). In addition, when the number of children was considered, women without children had higher job satisfaction than full-time workers (2). Aleksynska (2018)(1) showed that job satisfaction was higher for workers in couples and with children. In terms of workers’ age, a U-shaped relationship between age and job satisfaction has been recurrently reported and workers who are degree educated showed lower levels of job satisfaction (4).

Our study results indicate that women have lower job satisfaction than men. Furthermore, individuals who are not married or partnered report lower job satisfaction compared to those who are married or partnered. Additionally, our study finds that households with incomes below the median US household income reported higher job satisfaction than their counterparts. This result aligns with previous research that suggests a possible link between income and job satisfaction, with lower-paid workers potentially appreciating their jobs more and being more satisfied with them (5–7). Overall, our study highlights the importance of considering various demographic factors when examining job satisfaction.

*Table A1*. Regression results: Association between job satisfaction, work attendance anxiety, and precarious work schedules with demographic characteristics

| **Measures** | **Job Satisfaction** |
| --- | --- |
| **Work Attendance Anxiety** | -3.683*** |
|  | (0.630) |
| **Timing of hours** | -3.843** |
| Non-standard hours | (1.522) |
| **Predictability of Hours** | 1.339 |
| Less than a week notice | (1.687) |
| **Schedule Control of Hours** | -7.449*** |
| No employee input | (1.578) |
| **Work Hour Instability Ratio** | 2.267 |
|  | (1.430) |
| **Household Income** | 8.709*** |
| Below 75,000 | (1.993) |
| **Marital Status** | -6.803*** |
| Not married or not partnered | (2.140) |
| **Number of Children** | 2.926 |
| 1 and more | (2.090) |
| **Sex** | -2.943* |
| Female | (1.629) |
| **Race** | -0.881 |
| African American | (2.588) |
| **Age** | -0.030 |
|  | (0.072) |
| Observations | 179 |
| Adjusted R^2^ | 0.354 |

*Source:* Survey data collected by the authors.

*Notes:* Standard errors are presented in parentheses. *p<.05; **p<.01; ***p<.001

**S1 Appendix B. Detailed descriptive results on COVID-19 impact on businesses and workers**

Table B1 shows the descriptive statistics of COVID-19 impact on businesses and workers. Before presenting the results regarding job satisfaction and precarious work schedules, it is imperative to explore the repercussions of COVID-19. There has been a severe impact on businesses. Workers were calling out sick or afraid to come into work (54.7%) and staying at home to take care of their children or family (49.2%). Workers’ anxiety level was 2.66 on average with a standard deviation of 1.27. Workers with higher anxiety (48.4%) were more likely to work fewer hours after the COVID-19 outbreak than workers with lower anxiety (34.9%). The main reason for working fewer hours among workers with higher anxiety was that their employer had cut back hours (75.6%). Also, workers with higher anxiety were less provided with paid leaves (14%) and personal protective equipment (76.3%) than workers with lower anxiety (33.7%, 93% respectively).

*Table B1*. Descriptive statistics: Measures of COVID-19 impact on businesses and workers

|  | **Lower Anxiety** | **Higher Anxiety** | **Overall** |
| --- | --- | --- | --- |
| **Work Attendance Anxiety** |  |  |  |
| Mean (SD) | 1.51 (0.503) | 3.72 (0.713) | 2.66 (1.27) |
| **Impact on Businesses [Multi-select questions]** |  |  |  |
| Workers are calling out sick or are afraid to come into work | 48.8% | 60.2% | 54.7% |
| Workers are staying home to take care of children or family | 46.5% | 51.6% | 49.2% |
| Forced to close by government | 7.0% | 15.1% | 11.2% |
| Sales, revenue, business, or traffic is down | 39.5% | 48.4% | 44.1% |
| There has been no impact | 20.9% | 9.7% | 15.1% |
| Others | 8.1% | 6.5% | 7.3% |
| Don’t know | 1.2% | 0% | 0.6% |
| **Business Change [Multi-select questions]** |  |  |  |
| Cut back hours | 54.7% | 53.8% | 54.2% |
| Permanently shut down the business | 0% | 2.2% | 1.1% |
| Temporarily closed office, store, public location – but not the whole business | 12.8% | 23.7% | 18.4% |
| Temporarily closed the business, plans to reopen | 2.3% | 10.8% | 6.7% |
| No changes | 37.2% | 32.3% | 34.6% |
| Others | 7.0% | 5.4% | 6.1% |
| Don’t know | 1.2% | 0% | 0.6% |
| **Businesses Support [Multi-select questions]** |  |  |  |
| Provided flexible work schedules per worker’s request | 32.6% | 32.3% | 32.4% |
| Provided paid leaves if a worker was tested positive or have been directed | 33.7% | 14.0% | 23.5% |
| Provided personal protective equipment | 93.0% | 76.3% | 40.2% |
| Provided special bonus or increased hourly wages | 20.9% | 20.4% | 20.7% |
| Others | 1.2% | 2.2% | 1.7% |
| Don't know | 1.2% | 8.6% | 5.0% |
| **Work Hour Change** |  |  |  |
| I’m working less hours | 34.9% | 48.4% | 41.9% |
| I’m working more hours | 8.1% | 7.5% | 7.8% |
| I’m working the same hours | 57.0% | 43.0% | 49.7% |
| I’m not working at all | 0% | 1.1% | 0.6% |
| Observations | 86 | 93 | 179 |
| **Reason for Working Less Hours [Multi-select questions]** | |  |  |
| Another family member is currently working in my household | 6.7% | 6.7% | 6.7% |
| I am afraid to come into work | 6.7% | 13.3% | 10.7% |
| I am living in a household with someone who is at high risk from COVID-19 such as elderly or immune-compromised persons. | 3.3% | 11.1% | 8.0% |
| I have enough household income for daily necessities | 10.0% | 3.3% | 12.0% |
| My employer has cut back hours | 80.0% | 75.6% | 77.3% |
| Others | 10.0% | 4.4% | 6.7% |
| Observations | 30 | 45 | 75 |
| **Reason for Working Same or More Hours [Multi-select questions]** | |  |  |
| I am not afraid to come into work | 58.9% | 17.0% | 39.8% |
| I am the only family member who is employed and working in the household | 1.8% | 21.3% | 10.7% |
| I do not have enough household income for daily necessities | 30.4% | 53.2% | 40.8% |
| My employer asked me to work same/more hours | 50.0% | 46.8% | 48.5% |
| Others | 3.6% | 8.5% | 5.8% |
| Observations | 56 | 47 | 103 |

*Source:* Survey data collected by the authors on Amazon Mechanical Turk in July-August 2020.

**S1 Appendix C. Mediation models**

One might suggest that the positive effect of employer support on job satisfaction is mediated by a reduction in work attendance anxiety. Our mediation analysis indicates that work attendance anxiety partially mediates the relationship between two types of employer support—PPE (𝛽=2.16, p<0.05) and paid leave (𝛽=1.90, p<0.05)—and job satisfaction. Sobel's test confirms the significance of this mediation

Table C1. Mediation effect of work attendance anxiety on the relationship between employer support and job satisfaction

| Path | Path (descriptive) | Beta coefficient | | | |
| --- | --- | --- | --- | --- | --- |
|  |  | PPE | Paid Leave | Wage Increase | Flexible Work Schedule |
| c | Total effect of employer support on job satisfaction | 1.604 | 1.096 | 1.799 | 5.5133 |
| c’ | Direct effect of employer support on job satisfaction, controlling for work attendance anxiety and demographic characteristics | -0.56 | -0.807 | 1.05 | 4.592 |
| c’’ | Indirect effect of employer support on job satisfaction, controlling for work attendance anxiety and demographic characteristics | 2.165* | 1.904* | 0.749 | 0.921 |
| a | Effect of employer support on work attendance anxiety, controlling for demographic characteristics | -0.703 | -0.614 | -0.248 | -0.324 |
| b | Effect of work attendance anxiety on job satisfaction, controlling for employer support and demographic characteristics | -3.077 | -3.1 | -3.018 | -2.837 |

*Source:* Survey data collected by the authors.

*Notes:* Standard errors are presented in parentheses. Statistical significance is only denoted for the indirect effects. *p<.05; **p<.01; ***p<.001

Some might suggest a path analysis that precarious work schedules can lead to higher anxiety and thereby leading to lower job satisfaction. However, this is not supported in our analyses.

Table C2. Mediation effect of work attendance anxiety on the relationship between precarious work schedules and job satisfaction

| Path | Path (descriptive) | Beta coefficient | | | |
| --- | --- | --- | --- | --- | --- |
|  |  | Timing of hours | Predictability of Hours | Schedule Control of Hours | Work Hour Instability Ratio |
| c | Total effect of precarious work schedules on job satisfaction | -4.572 | -0.589 | -5.057 | 1.695 |
| c’ | Direct effect of precarious work schedules on job satisfaction, controlling for work attendance anxiety and demographic characteristics | -3.427 | -0.436 | -5.503 | 2.417 |
| c’’ | Indirect effect of precarious work schedules on job satisfaction, controlling for work attendance anxiety and demographic characteristics | -1.144 | -0.153 | 0.446 | -0.722 |
| a | Effect of precarious work schedules on work attendance anxiety, controlling for demographic characteristics | 0.404 | 0.05 | -0.141 | 0.231 |
| b | Effect of work attendance anxiety on job satisfaction, controlling for employer support and demographic characteristics | -2.829 | -3.041 | -3.161 | -3.116 |

*Source:* Survey data collected by the authors.

*Notes:* Standard errors are presented in parentheses. Statistical significance is only denoted for the indirect effects. *p<.05; **p<.01; ***p<.001

**S1 Appendix D. Impact of proxy for fear of transmission during COVID-19 on job satisfaction**

Although we did not directly measure the fear of COVID-19 infection, we inquired whether employees were calling out sick or afraid to come into work, which can imply a fear of transmission. Using this proxy, our regression results show that fear of transmission is negatively associated with job satisfaction, similar to work attendance anxiety.

Table D1. Impact of proxy for fear of transmission during COVID-19 on job satisfaction

| **Measures** | **Job Satisfaction** | | | | |
| --- | --- | --- | --- | --- | --- |
|  | (1) | (2) | (3) | (4) | (5) |
|  |  |  |  |  |  |
| **Work Attendance Anxiety** |  |  | -3.299*** | -3.469*** | -3.424*** |
|  |  |  | (0.685) | (0.674) | (0.636) |
|  |  |  |  |  |  |
| **Business Impact: Employees are calling out sick or are afraid to come into work** | -4.269** | -4.936^***^ | -3.071* | -4.113** | -3.802** |
|  | (1.802) | (1.699) | (1.713) | (1.677) | (1.586) |
| **Timing of hours** |  |  |  | -3.626** | -3.468** |
| Non-standard hours |  | -4.068^**^ |  | (1.659) | (1.545) |
|  |  | (1.665) |  |  |  |
| **Predictability of Hours** |  | 0.817 |  | 1.574 | 1.73 |
| Less than a week notice |  | (1.825) |  | (1.676) | (1.697) |
| **Schedule Control of Hours** |  | -8.035^***^ |  | -7.126*** | -8.105*** |
| No employee input |  | (1.724) |  | (1.665) | (1.596) |
| **Work Hour Instability Ratio** |  | 1.763 |  | 1.467 | 2.221 |
|  |  | (1.494) |  | (1.426) | (1.385) |
| Observations | 179 | 179 | 179 | 179 | 179 |
| Adjusted R^2^ | 0.138 | 0.248 | 0.237 | 0.232 | 0.356 |
| Demographic Controls | Yes | Yes | Yes | No | Yes |

*Source*: Survey data collected by the authors.

*Notes:* Standard errors are presented in parentheses. Demographic control variables include household income, marital status, number of children, sex, race, and age. *p<.05; **p<.01; ***p<.001

**References**

1. Aleksynska M. Temporary employment, work quality, and job satisfaction. Journal of Comparative Economics. 2018;46(3):722–35.

2. Booth AL, Van Ours JC. Job Satisfaction and Family Happiness: The Part‐Time Work Puzzle. The Economic Journal. 2008 Feb 1;118(526):F77–99.

3. Andrea SB, Eisenberg-Guyot J, Oddo VM, Peckham T, Jacoby D, Hajat A. Beyond Hours Worked and Dollars Earned: Multidimensional EQ, Retirement Trajectories and Health in Later Life. Wang M, editor. Work, Aging and Retirement. 2022 Jan 6;8(1):51–73.

4. Perales F, Tomaszewski W. Happier with the Same: Job Satisfaction of Disadvantaged Workers: Happier with the Same: Job Satisfaction. British Journal of Industrial Relations. 2016 Dec;54(4):685–708.

5. Binder M, Coad A. Life satisfaction and self-employment: a matching approach. Small Bus Econ. 2013 May 1;40(4):1009–33.

6. Judge TA, Watanabe S. Another look at the job satisfaction-life satisfaction relationship. Journal of Applied Psychology. 1993;78(6):939–48.

7. Kim H, Stoner M. Burnout and Turnover Intention Among Social Workers: Effects of Role Stress, Job Autonomy and Social Support. Administration in Social Work. 2008 Jun 13;32(3):5–25.
